# Respiratory viruses within homeless shelters in Marseille, France

**DOI:** 10.1186/1756-0500-7-81

**Published:** 2014-02-05

**Authors:** Simon-djamel Thiberville, Nicolas Salez, Samir Benkouiten, Sekene Badiaga, Remi Charrel, Philippe Brouqui

**Affiliations:** 1 UMR190, Faculty of medecine "Emergence des Pathologies Virales" (Aix-Marseille Univ. – IRD French Institute of Research for Development – EHESP French School of Public Health, Marseille, France), 27 bd Jean MOULIN, Marseille 13005, France; 2 Faculté de médecine, Institut Hospitalo-Universitaire des Maladies Infectieuses et Tropicales URMITE CNRS IRD UMR 6236/198, 27 bd Jean MOULIN, Marseille 13005, France

**Keywords:** Respiratory tract disease, Influenza virus, Rhinovirus, Metapneumovirus, Coronavirus, Respiratory syncytial virus, Asymptomatic infections, Homeless persons

## Abstract

**Background:**

Homeless shelters are identified as places where humans are at high risk of acquiring respiratory disease. We previously reported the prevalence of the main respiratory diseases affecting a population of homeless in Marseille, France. Here, we investigated the prevalence of 10 respiratory viruses in a similar homeless population during 2 successive winter seasons.

**Findings:**

Following a clinical examination, we collected nasal specimens from which the RT-PCR detection of 10 respiratory viruses was performed through snapshot investigations. Among the 265 patients included, 150 (56.6%) reported at least one respiratory symptom of which 13 (8.7%) had positive swabs for at least one respiratory virus, and 115 patients reported any respiratory symptom of which 10 (8.7%) had positive swabs for respiratory virus. Overall, 23 patients had positive swabs for at least one respiratory virus. Human rhinovirus (HRV) was the predominant virus (13 isolates) followed by enteroviruses (3), human metapneumovirus (2), human coronavirus OC43 (2), 229E virus (2) and human respiratory syncytial virus subtype B (1). Among the patients infected with HRV, 10 were collected during the same snapshot.

**Conclusions:**

Although one half of the patients reported respiratory symptoms, the prevalence of respiratory viruses was within the range of that previously described in adult asymptomatic patients outside the homeless community. Most HRV-positive swabs were collected during the same snapshot suggesting a local outbreak. No influenza viruses were found despite the fact that one half of the patients were investigated during the peak of the seasonal influenza epidemic in Marseille.

## Background

The homeless are defined as people who do not have customary and regular access to a conventional dwelling or residence [1]. They have significantly higher rates of underlying illnesses (chronic obstructive pulmonary disease, alcoholism) that predisposes them to a variety of diseases, particularly respiratory diseases [2]. Moreover, homeless shelters are identified as places where persons are at high risk of acquiring respiratory disease [3].

Nevertheless, the prevalence and transmission of influenza and other respiratory viruses among homeless populations are currently poorly studied [4,5].

In Marseille, France, there are an estimated 1,500 homeless persons, of which approximately 600 regularly use the 2 main shelters. Since 1993, we have studied homeless populations through snapshot interventions [4]. In 2005, we reported on the prevalence of the main respiratory pathogens and diseases affecting this population [4].

Here, we investigated the prevalence of respiratory viruses of homeless people in shelters in Marseille, during 2 successive winter seasons.

## Methods

The study protocol was approved by the Ethical Committee of the School of Medicine of Aix-Marseille University under n° 10-005. This study was conducted on February 1st and February 4th, 2010 (first snapshot) and on February 1st and February 3rd, 2011 (second snapshot) in 2 homeless shelters (designated A and B) in Marseille, France, as previously described [4].

After written informed consent for participation, homeless persons were interviewed and examined by a medical doctor. Thereafter, nasal specimens were collected with a 2-ml MW950S virocult swab (Sigma, Wiltshire, U.K.). Ten respiratory viruses were tested using real-time RT-PCR [6]: influenza virus A, B and A/2009/H1N1, respiratory syncytial viruses A and B (RSV-A, RSV-B) human coronavirus (hCoV) OC43 and E229, human rhinovirus (HRV), enteroviruses (EV) and human metapneumovirus (hMPV).

Epidemiologic, clinical and laboratory data were analysed with SPSS 20.0 (SPSS Inc., Chicago, IL, USA). Two-tailed tests were used for comparisons. Differences in proportions were tested using Fisher’s exact test. Continuous variables were tested using the Mann-Whitney non-parametric test. Statistical significance was set at p & 0.05.

## Findings

**Figure 1:**
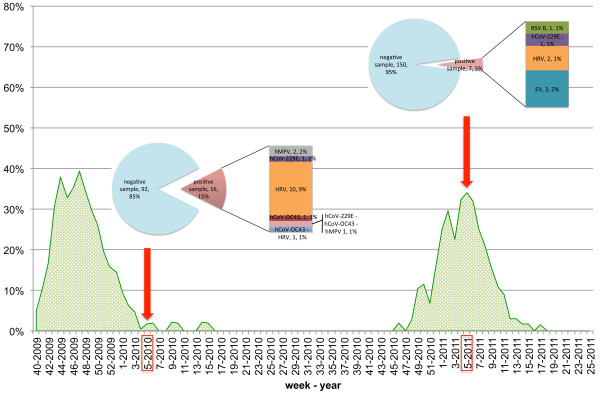
**Periods of the 2 snapshot investigations (red arrows) in relation to the weekly circulation of influenza based on the percentage of positive influenza samples received at the diagnostic laboratory of the University Hospital of Marseille (green curve).** The pie charts represent the percentage of positive respiratory virus found during the 2 snapshot investigations.

The 2 snapshots were positioned temporally at different periods of local peaks of influenza virus circulation as assessed by the diagnostic laboratory of the University Hospital of Marseille (Figure 1).

Two hundred and sixty-five (108 in 2010 and 157 in 2011) homeless persons were included (representing approximately 22% of the homeless people living in the 2 main shelters of Marseille). The demographic characteristics and underlying disease conditions were not significantly different in 2010 and 2011 (Table 1).

**Table 1:** Demographic characteristics of the study population

**Characteristics**	**2010**	**2011**
	**n/N (%)**	**n/N (%)**
Age		
Mean, standard deviation (SD)	48.8, 17.4	46.7, 16.8
Sex		
Men	95/108 (88.0)	142/157 (90.4)
Women	13/108 (12.0)	15/157 (9.6)
Shelters		
A	73/95 (76.8)	85/157 (54.1)
B	22/95 (23.2)	72/157 (45.9)
Addiction		
Current smoker	67/105 (62.0)	90/155 (58.1)
Alcohol abuser	24/105 (22.2)	32/155 (20.6)
Marijuana use	16/105 (14.8)	28/155 (18.1)
Injection use	1/105 (1.0)	5/155 (3.2)
Inhaled drug	3/105 (2.8)	8/155 (5.2)
Underlying disease		
Tuberculosis	9/96 (9.4)	6/151 (4.0)
Chronic bronchitis	12/108 (11.1)	11/157 (7.0)
Asthma	7/108 (6.5)	14/157 (8.9)
Cancer	3/108 (2.8)	4/157 (2.5)
Diabetes	5/108 (4.6)	11/157 (7.0)
Chronic hepatitis	4/108 (3.7)	5/157 (3.2)

Among the 265 patients, 23 (8.7%) were found positive for at least one virus (16 in 2010 and 7 in 2011). In 2 cases (2/23, 8.7%), more than one virus was detected (1 patient with hCoV-OC43, hCoV-229E and hMPV, and 1 patient with hCoV-OC43 and HRV). Three patients were positive for EV, while hMPV, hCoV-OC43, hCoV-229E and RSV B were isolated in 2, 2, 2 and 1 sample, respectively. No influenza virus was detected. HRV was detected in 13 of the 23 positive samples (56.5%). Ten of these 13 HRV-positive samples were collected during the same snapshot on February 1, 2010 in shelter A, representing a prevalence of 11.6% in this shelter (Figure 1).

Among the 265 patients, fever was observed in 1 (0.4%) patient. Headache, arthralgia and myalgia were recorded in 31.7% (83/262), 36.2% (94/260) and 20.5% (53/258), respectively. At least one respiratory symptom (cough, sputum production, dyspnoea, odynophagia or abnormal pulmonary auscultation) was identified in 56.6% (150/265) of the cases with a similar proportion in 2010 (59.3%) and 2011 (54.8%).

Among the 150 patients with at least one respiratory symptom, 13 (8.7%) had positive swabs for at least one virus (7 HRV, 2 EV, 1 VRS-B, 1hCoV-229E, 1 hCoV-OC43, and 1 patient with a co-infection of HRV/hCoV-OC43). Among the remaining 115 patients who did not report any respiratory symptoms, 10 (8.7%) had positive swabs for at least one respiratory virus (5 HRV, 1 EV, 2 hMPV, 1 hCoV-229E, and 1 patient with a co-infection of hCoV-OC43/ hCoV-229E/ hMPV).

## Discussion

We describe the prevalence of respiratory viruses amongst homeless people in Marseille, France. A total of 8.7% of homeless persons tested positive for at least one respiratory virus. Although one half of the patients reported at least one respiratory symptom, this prevalence was not drastically different from that observed in adult asymptomatic patients (range between 4 and 17%) [7-9].

HRV was the most frequently virus identified in agreement with previous studies that investigated respiratory viruses in asymptomatic patients [9,10]. Interestingly, HRV-positive patients were sampled in one shelter during the same snapshot, suggesting a local outbreak. Local transmission of HRV was also investigated in a household study which highlighted that during a local transmission of HRV, adult were often asymptomatic [11].

Interestingly, none of the samples were positive for influenza virus despite the fact that the 2011 snapshot (representing 59.2% [157/265] of studied persons) was conducted during the peak period of the local influenza outbreak (Figure 1). Moreover, among the homeless people included during the 2011 snapshot, the rate of seasonal influenza vaccination was reported as 35.6% (data not shown), which was lower than the estimated influenza vaccination coverage in persons aged less than 65 years with an underlying condition in France in January 2011 (46.6%) [12]. Recently, a study suggested that the social isolation of homeless people might have been protective against pandemic influenza 2009 [5].

Although this snapshot investigation was limited in its scope, our data support the hypothesis that the isolation of homeless people from the general population, notably children who play a major role in respiratory virus transmission, might have a protective impact against community respiratory viruses such as influenza virus [13]. Further longitudinal investigations within homeless shelters are needed.

## Abbreviations

EV: Enterovirus; hCoV: Human coronavirus; hMPV: Human metapneumovirus; HRV: Human rhinovirus; RSV-A: RSV-B, respiratory syncytial viruses A and B.

## Competing interests

The authors declare that they have no competing interests.

## Authors’ contributions

Conceived and designed the experiments: PB, SBa, SBe. Performed the investigations: PB, SBa, SBe, SDT. Analyzed the data: PB, RC, SDT. Contributed reagents/materials/analysis tools: NS, PB, RC, SBA, SBE, SDT. Wrote the paper: PB, RC, SDT. All authors read and approved the final manuscript.
